# Design of a Concentric Multi-Scale Zoom Optical System Based on Wide Object Distance and High-Precision Imaging

**DOI:** 10.3390/s22197356

**Published:** 2022-09-28

**Authors:** Kun Zhang, Zheng Qu, Jingchen Li, Jian Wang, Si Sun, Fan Yang

**Affiliations:** 1 Institute of Optics and Electronics, Chinese Academy of Sciences, Chengdu 610209, China; 2 Changchun Institute of Optics, Fine Mechanics and Physics, Chinese Academy of Sciences, Changchun 130033, China; 3 University of Chinese Academy of Sciences, Beijing 100049, China

**Keywords:** concentric multi-scale optical system, zoom optical system, image stitching misalignment, large field of view, high resolution

## Abstract

To effectively balance the trade-off between a large field of view (FOV) and high resolution of an optical system, as well as to solve the problem of image stitching misalignment after focusing, firstly, this paper conducts a theoretical analysis of the design principle of the concentric multi-scale optical system and the causes of image stitching misalignment after focusing. Secondly, the design idea of using a combination structure of a two-layer front concentric imaging group and an image-space telecentric relay imaging array and then a joint full-motion zoom relay imaging system is proposed. Finally, an image-space telecentric two-step zoom concentric multi-scale optical system with a 7 × 7 relay imaging array is designed. The FOV of this optical system is 60° × 45°; the focal lengths are 50 mm and 100 mm for the center channel and 50 mm for the other channels. This concentric multi-scale zoom system has the advantages of both high-precision imaging stitching with a wide object distance and high-resolution imaging, which makes up for the defects of the conventional concentric multi-scale optical system, making it a promising application in the fields of aviation and security.

## 1. Introduction

With the advancement of information technology, people are becoming more interested in acquiring more detailed information from the external environment. The amount of information obtained by an optical system is chiefly determined by two factors: field of view (FOV) and resolution [1,2], where a large FOV can cover a wide observation range and a high resolution can help to capture more detailed information about the observed object. However, there is a mutually restrictive relationship between these two factors, and to balance the trade-off between a large FOV and high resolution, the concentric multi-scale optical system was born [3,4,5]. The imaging advantages of concentric multi-scale optical systems make them promising for a wide range of applications in national defense construction, security monitoring, unoccupied aerial vehicle (UAV) reconnaissance, and remote sensing mapping [6,7,8].

Brady et al. first proposed a concentric multi-scale optical imaging system [9,10], in which each secondary relay imaging group in the system is identical, and the local aberrations corrected by the secondary relay imaging groups at different field-of-view locations are also identical; they developed three prototypes: AWARE-2 [6,11], AWARE-10 [12], and AWARE-40 [13]. The front system of AWARE-2 is a double-layer concentric spherical lens, the secondary system is a relay imaging array of 98 micro-cameras, and the combined focal length of the entire system is 34.2 mm with an F-number of 2.1 and a FOV of 120° × 50°. The front system of AWARE-10 is a double-layer concentric spherical lens, the secondary system is a relay imaging array of 382 micro-cameras, and the combined focal length of the overall optical system is 53.21 mm with an F-number of 3.2 and a FOV of 100° × 60°. AWARE-40 has a non-concentric double Gaussian objective lens for the front system to increase the focal length of the objective lens of the front system, and the secondary system is a relay imaging array of 262 micro-cameras, the combined focal length of the optical system is 130 mm, the F-number is 3.6, and the full FOV is 36°. Wu et al. [14] developed a principle prototype of a concentric multi-scale optical system with 3 × 3 relay cameras, with a combined focal length of 17 mm, an F-number of 2.8, and a full FOV of 70°.

At present, to realize the perfect stitching of concentric multi-scale panoramic images, the traditional approach is to use the stitching scheme of feature point extraction and recognition [15,16], which is independent of the change of imaging object distance of the optical system and is mainly closely related to the matching number of feature points of adjacent images. However, this severely restricts the application scene of imaging, such as frequent problems with the insufficient number of matching single background feature points and stitching errors. Additionally, traditional stitching methods are also very time-consuming, and stitching for billions of pixels is difficult to satisfy the demand of real-time stitching imaging applications [17,18]. Therefore, in order to solve the problems of single background stitching accuracy and stitching efficiency, point-to-point stitching imaging with a concentric multi-scale optical system was developed to solve the issues of single background stitching accuracy and stitching efficiency. This method directly stitches the overlapping pixel positions of adjacent images by calibrating the internal parameters of the camera, thus reducing the stitching steps and significantly improving the stitching efficiency, but the stitching method is very dependent on the internal parameters of the optical system. Consequently, this paper investigates the design method of the concentric multi-scale optical system focusing on the stability of the internal parameters within a wide object distance range.

This paper proposes a concentric multi-scale zoom optical system design method based on wide object distance and high-precision imaging for addressing the above problems. The front concentric imaging group is designed by a two-layer concentric spherical lens structure, and the relay imaging array is designed with a telecentric structure in image space to realize the entire concentric multi-scale optical system with a wide object distance and high-precision imaging, as well as to solve the problem of stitching misalignment of the imaging after focusing. To further improve the resolution of the optical system to obtain more detailed information about the target of interest, a design method of an image-space telecentric two-step zoom relay imaging group is proposed. Finally, to verify the feasibility of this design method, an image-space telecentric two-step zoom concentric multi-scale optical system is designed.

## 2. Principle

### 2.1. Principle of the Initial Structure Calculation

Generally, a concentric multi-scale optical system mainly consists of a front concentric imaging group and a secondary relay imaging group array [19]. The front concentric imaging group is a two-layer or three-layer concentric collodion spherical lens group, which is rotationally symmetric and has no main optical axis in the usual sense, thus the aberration associated with the FOV is small and can achieve a large field-of-view imaging and high-efficiency energy collection. The relay imaging group arrays are arranged at a certain distance behind the front concentric imaging group, the FOV of the front concentric imaging group is divided, and there is an overlap between the FOVs of adjacent channels, which provides a guarantee for image stitching. Furthermore, the relay imaging group can also further correct the residual aberration of the front concentric imaging group, solving the problem of the mutual constraints of FOV and resolution of the conventional single-aperture imaging system, thus achieving a large FOV and high-resolution imaging at the same time. To simplify the complexity of the relay imaging group array, each relay imaging group is identical, at which point the full FOV of the entire system has the same resolution [20]. A theoretical analysis of the initial structure calculation method of the concentric multi-scale optical system will be shown in the next part.

**Figure 1:**
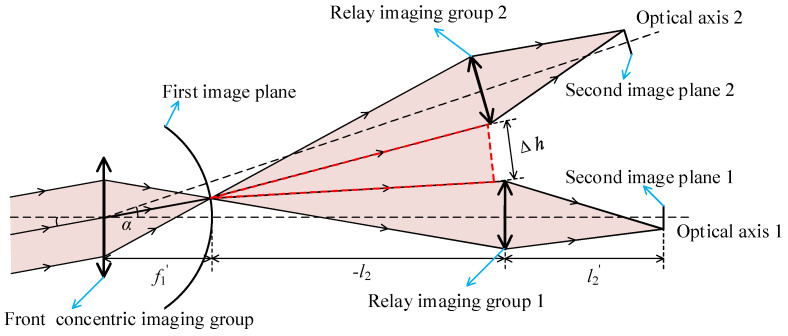
Imaging principle diagram of a concentric multi-scale optical system.

From the imaging principle diagram of a concentric multi-scale optical system (shown in Figure 1), the total length of this system is

(1) $$l=f_{1}{}^{\prime }-l_{2}+l_{2}{}^{\prime }$$

The magnification of the relay lens group is

(2) $$\beta =\frac{l_{2}{}^{\prime }}{l_{2}}$$

The focal length of the concentric multi-scale optical system is

(3) $$f^{\prime }=\beta f_{1}{}^{\prime }$$

The image-space numerical aperture *NA*_1_^′^ of the front concentric imaging group can be expressed as

(4) $$NA_{1}{}^{\prime }=n_{1}{}^{\prime }\sin\theta_{1}{}^{\prime }=n_{1}{}^{\prime }\times \frac{D_{1}/2}{\sqrt{f_{1}{{}^{\prime }}^{2}+{\left(D_{1}/2\right)}^{2}}}=n_{1}{}^{\prime }\times \frac{1}{\sqrt{4F_{1}{}^{2}+1}}$$

where *n*_1_^′^ and *θ*_1_^′^ are the respective refractive index and aperture angle of the front imaging group in the imaging space, *D*_1_ is the pupil diameter of the front concentric imaging group, *f*_1_^′^ is the focal length of the front concentric imaging group, and *F*_1_ is the F-number of the front concentric imaging group.

The image-space numerical aperture *NA*^′^ of the entire concentric optical system is

(5) $$NA^{\prime }=n^{\prime }\sin\theta^{\prime }=n^{\prime }\times \frac{D/2}{\sqrt{f^{\prime }{}^{2}+{\left(D/2\right)}^{2}}}=n^{\prime }\times \frac{1}{\sqrt{4F^{2}+1}}$$

where *n*^′^ and *θ*^′^ denote the refractive index and aperture angle of the image-space of the concentric multi-scale optical system, *D* denotes the pupil diameter of the concentric multi-scale optical system, *f*_1_^′^ denotes the focal length of the concentric multi-scale optical system, and *F* denotes the F-number of the concentric multi-scale optical system.

When the concentric multi-scale optical system has no vignetting, the mechanical gap Δh between relay imaging group 1 and relay imaging group 2 can be expressed as

(6) $$\Delta h=-2l_{2}\times \tan\left[\theta_{1}{}^{\prime }+2\arctan\left(\frac{\tan\theta^{\prime }}{\beta }\right)\right]$$

In the design of a concentric multi-scale optical system, the mechanical gap between relay imaging group 1 and relay imaging group 2 must be greater than zero to guarantee sufficient mechanical space. The mechanical gap $\Delta h^{\prime }$ is defined for the required assembly of relay imaging groups 1 and 2 as

(7) $$\Delta h^{\prime }=-2l_{2}\times \tan\left[\theta_{1}{}^{\prime }+2\arctan\left(\frac{\tan\theta^{\prime }}{\beta }\right)\right]$$

When the concentric multi-scale optical system has no vignetting, the largest rotational angle $\alpha_{1}$ between optical axis 1 and optical axis 2 is

(8) $$\alpha_{1}=2\theta_{1}{}^{\prime }+2\arctan\left(\frac{\tan\theta^{\prime }}{\beta }\right)$$

The rotational angle α is the angle between the optical axes of adjacent relay imaging groups, which need to meet $\alpha_{1}\geq \alpha$ when the system has no vignetting.

(9) $$\alpha =\arctan\left(\frac{Ln-2L\eta m}{2f^{\prime }}\right)$$

where *L* is the long or short side of the detector sensing plane, *n* and *m* are positive integers, *n* ≥ 2 and *n* − *m* = 1, and is the field-of-view overlap rate of adjacent relay imaging groups, which needs to meet η≥5% for better image stitching. For instance, the *n* and *m* of the relay imaging group 1 and the relay imaging group 2 (seen in Figure 1) are equal to 2 and 1, respectively.

In summary, the initial structural parameters of the front concentric imaging group and the relay imaging group can be calculated by Equations (1)–(9).

### 2.2. Image Stitching Misalignment Analysis

To obtain a complete image, the images formed from each channel of the concentric multi-scale optical system need to conduct aberration correction, image stitching, and image cropping processes [16]. Image stitching in concentric multi-scale optical systems is mainly divided into two methods: the first is to achieve image stitching by the feature point matching method [21], which requires sufficient feature matching points for adjacent overlapping images and is time-consuming; the second is to perform image stitching by point-to-point mapping relationships of adjacent overlapping images, which does not require feature matching points and it is fast, so it is the mainstream method of image stitching in concentric multi-scale optical systems.

**Figure 2:**
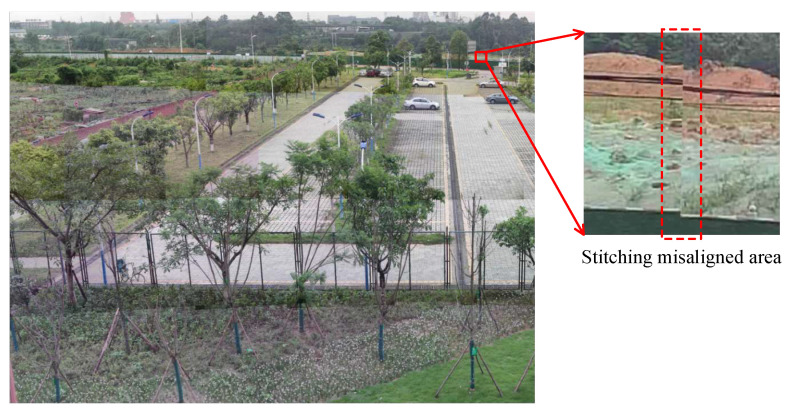
Stitching of the misaligned image.

Before the point-to-point mapping method for high-precision image stitching is used, the internal and external parameters of the optical system need to be strictly calibrated, and then the calibrated parameters are used for image stitching. However, the calibration of the internal parameters of the optical system is to calibrate the internal parameters of the optical system at a certain object distance [22], and the internal parameters will change after the optical system is focused, so when the previously calibrated parameters are used for image stitching, the stitched images will be mismatched. From the stitching misaligned image (illustrated in Figure 2) after focusing, it is obvious to find the mismatched phenomenon in the stitching misaligned area. The cause of the phenomenon will be analyzed in the following paragraphs.

**Figure 3:**
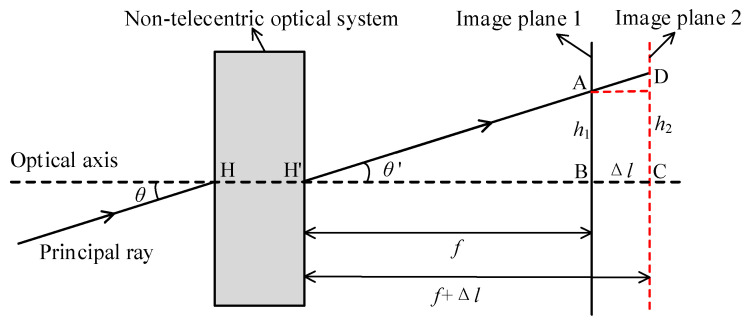
Imaging principle diagram of the non-telecentric optical system.

The imaging principle diagram of a non-telecentric optical system [23] is illustrated in Figure 3, which indicates that parallel light with an incident angle *θ* of passes through the optical system and is imaged at point A on image plane AB, with image height *h*_1_. When imaging an object from a limited distance, the focusing value of the optical system is Δ*l*, and the image is imaged at point D on the image plane after focusing, and the corresponding image height is *h*_2_.

**Figure 4:**
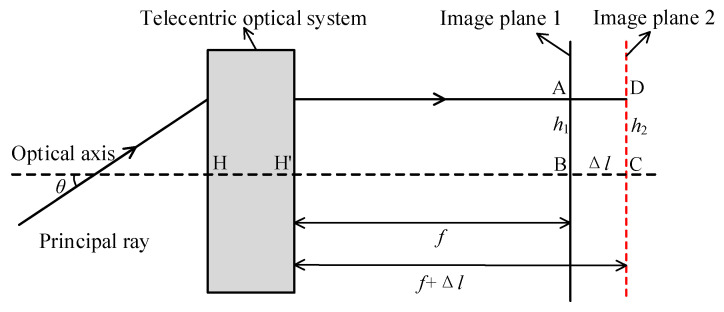
Imaging principle diagram of the telecentric optical system.

Figure 3 illustrates that the range of image height is $\Delta h^{\prime }$ when the optical system is focused on the same FOV θ. Consequently, when the previous calibration parameters are used for image stitching, the stitched images will be misaligned after focusing. To avoid the mismatch, two approaches can be used; the first approach is to re-calibrate after each focus, which is very restricted in practical implementation. The second approach is to design the optical system in such a way that the following Equation (10) $\theta^{\prime }=0$. The imaging principle diagram of a non-telecentric optical system is illustrated in Figure 4.

(10) $$\Delta h^{\prime }=\Delta l\times \tan\theta^{\prime }$$

As a result, this paper proposes employing an image-space telecentric structure to accomplish wide object distances and high-quality imaging, as well as to resolve the issue of high-precision image stitching.

## 3. Design and Results

### 3.1. Concentric Multi-Scale Optical System Design

#### 3.1.1. Design Specifications

In this paper, the specifications of the concentric multi-scale optical system are listed in Table 1. A 1/2-inch detector with a pixel size of 3.45 µm × 3.45 µm is selected, corresponding to a Nyquist frequency of 145 lp/mm. To satisfy the image stitching requirements of the concentric multi-scale optical system, the detector of adjacent optical channels has an overlapping rate of 5% in both the long-edge and short-edge directions.

#### 3.1.2. Design of the Front Concentric Imaging Group

The front concentric imaging group is typically designed with a two-layer or three-layer structure. The two-layer front concentric imaging group has a limitation of aberration correction capability, but the construction is relatively simple and the manufacturing cost is low. The three-layer front concentric imaging group has a higher aberration correction capability; however, the construction is more complicated to assemble. Hence, this paper employs a two-layer construction to design the front concentric imaging group.

**Figure 5:**
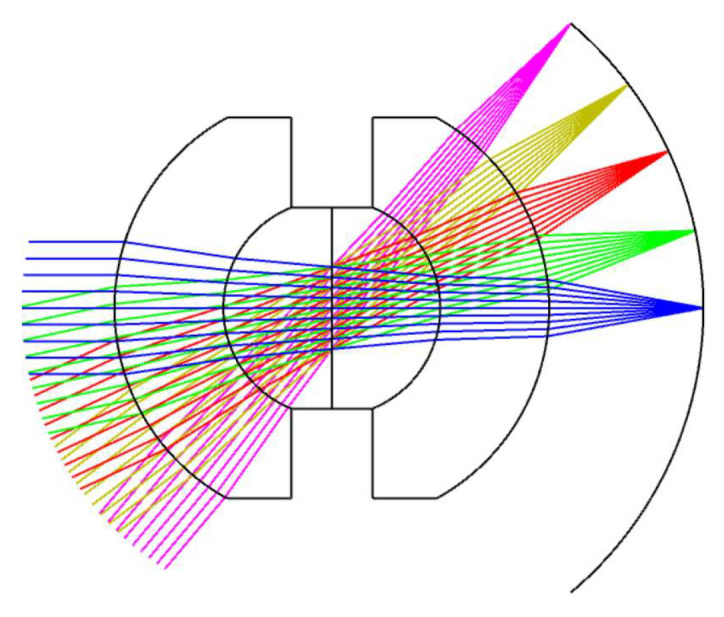
Configuration of the front concentric imaging group.

After the calculation for the initial configuration, the focal length of the front concentric imaging group is determined as 120 mm, and the F-number is 2.8. The front concentric imaging lens group with a two-layer structure (illustrated in Figure 5) is designed via the optical design software Zemax. To correct chromatic aberrations, two glass materials (H-ZLAF75A, H-ZK10L) are applied in the front concentric imaging group.

**Figure 6:**
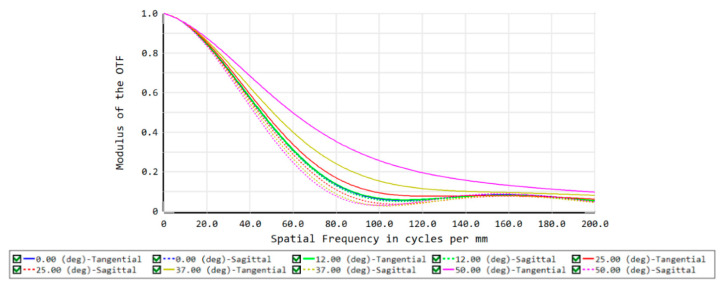
MTF curves of the front concentric imaging group.

**Figure 7:**
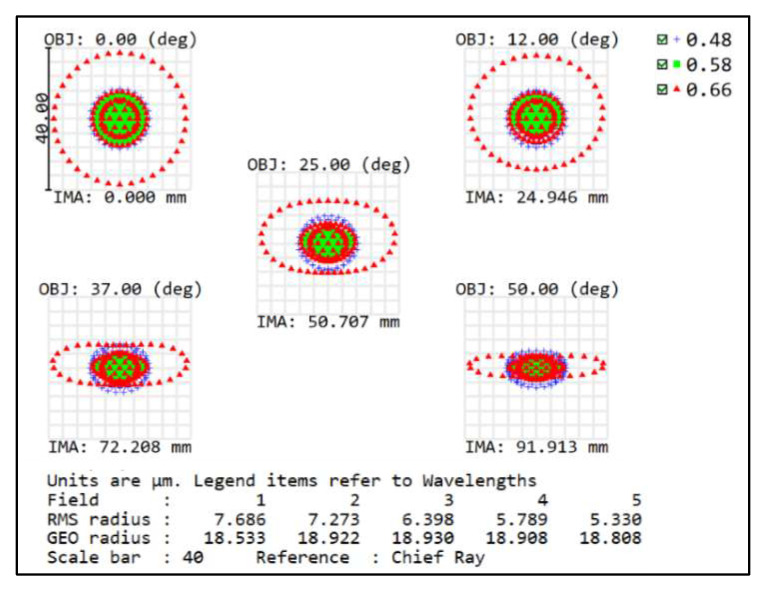
Spot diagram of the front concentric imaging group.

In the imaging-quality evaluation of an optical system, the modulation transfer function (MTF) and spot diagram are often shown to reflect the results. Therefore, the imaging quality of the front concentric imaging group is evaluated by the MTF (seen in Figure 6) and spot diagram (seen in Figure 7). The curves indicate that aberration correction is not ideal and it is urgent to connect the relay imaging group to compensate for the aberrations.

#### 3.1.3. Design of the Image-Space Telecentric Relay Imaging Group and Entire System

**Figure 8:**
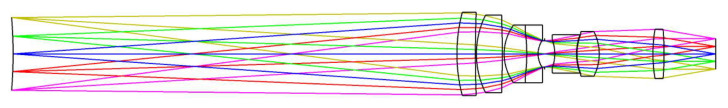
Image-space telecentric relay imaging group layout.

In the concentric multi-scale optical system, there are two main roles of the relay imaging group: the first is to perform secondary imaging and determine the focal length of the entire system together with the front concentric imaging group; the second is to compensate for the residual aberrations generated by the front concentric imaging group to improve the imaging quality of the optical system. To solve the problem that the internal parameters of the optical system change after focusing, the relay imaging group should adopt the image-space telecentric structure (shown in Figure 8) with the magnification *β* of −0.417.

**Figure 9:**
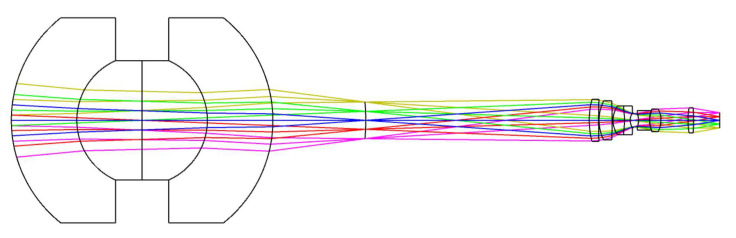
Image-space telecentric concentric multi-scale optical system layout.

By combining the two components of the front concentric imaging group and the relay imaging group, the final concentric multi-scale optical system is acquired, as illustrated in Figure 9. Depending on the focal length of the front concentric imaging group and the magnification of the relay imaging group, the focal length of the image-space telecentric concentric multi-scale optical system can be derived as 50 mm.

**Figure 10:**
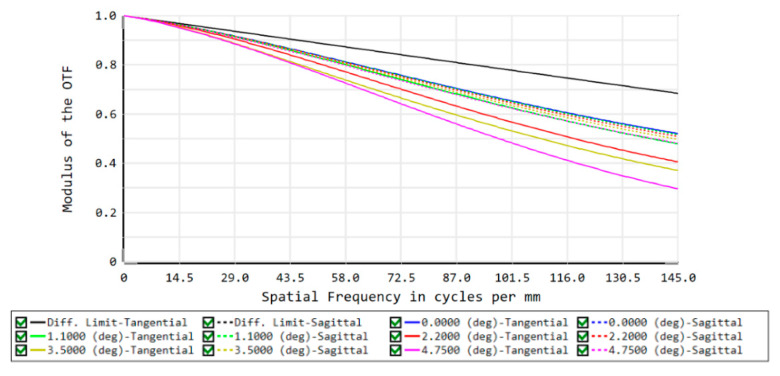
MTF curves of the concentric multi-scale optical system.

**Figure 11:**
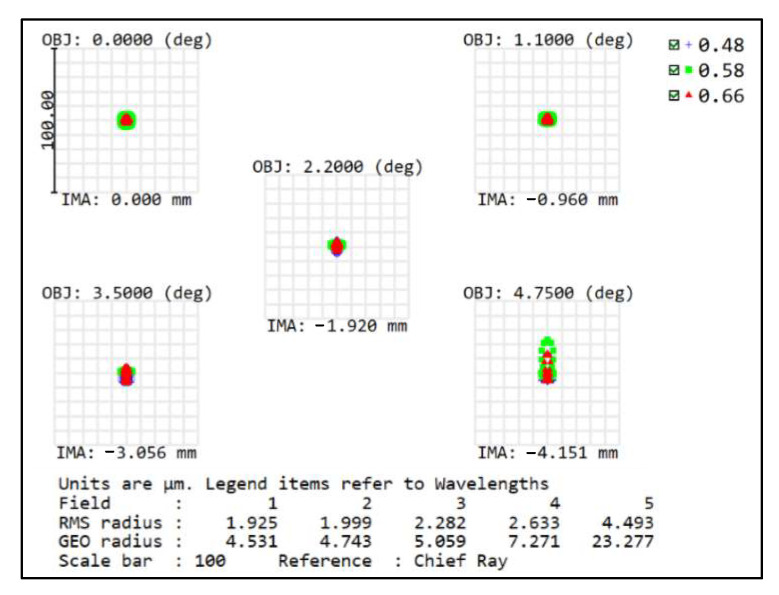
Spot diagram of the concentric multi-scale optical system.

**Figure 12:**
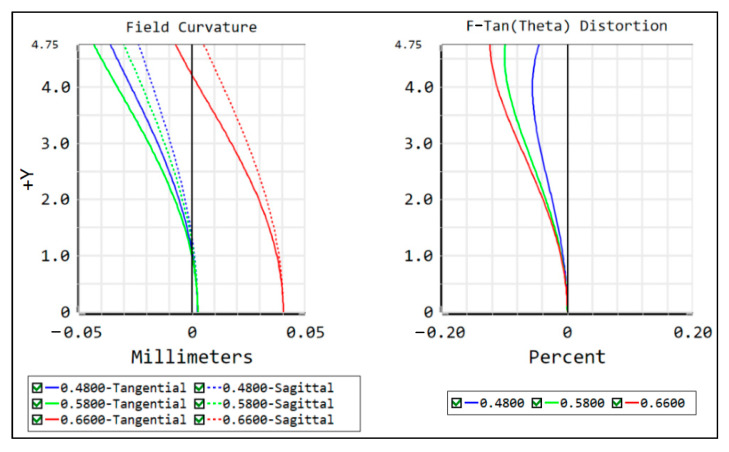
Field curvature and distortion curves of the concentric multi-scale optical system.

The MTF curve (in Figure 10), spot diagram (in Figure 11), field curvature, and distortion (in Figure 12) of the image-space telecentric concentric multi-scale optical system are simulated to evaluate the image quality, from which the MTF values of the optical system center FOV and edge FOV at the Nyquist frequency of 145 lp/mm are greater than 0.5 and 0.3, respectively. Generally, F-Theta distortion is used to characterize image deformation; the maximum distortion (illustrated in Figure 12) is lower than 0.15% and thus the optical system has excellent imaging quality.

### 3.2. Design of the Zoom Concentric Multi-Scale Optical System

#### 3.2.1. Design of the Telecentric Relay Imaging Group with Variable Magnification

To further improve the spatial resolution of the concentric multi-scale optical system, we propose replacing the relay imaging group with fixed magnification in the central channel with a variable magnification relay imaging group with adjustable magnification. To ensure that the images of the concentric multi-scale optical system can still be stitched together completely, the short focal length is determined to be 50 mm. To ensure that the image stitching will not be misalignment after focusing, the short focal length of the relay imaging group must be the telecentric configuration. When the focal length is 100 mm, the spatial resolution of the optical system doubles; however, the imaging FOV becomes smaller, and the image of the central channel optical system is not involved in image stitching at this time. Consequently, the short focal length of the zoom concentric multi-scale optical system is 50 mm and the long focal length is 100 mm, thus achieving two zoom levels.

**Figure 13:**
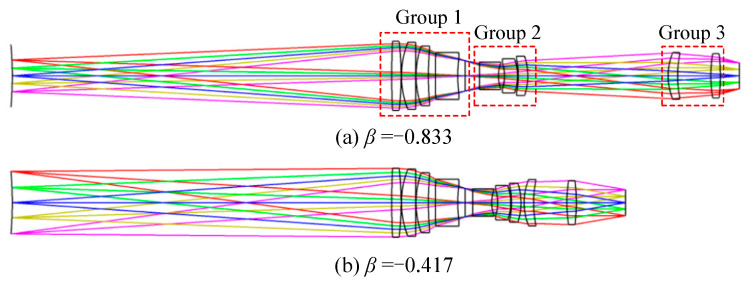
Configuration diagrams of the zoom relay imaging group. (**a**) magnification *β* = −0.833, (**b**) magnification *β* = −0.417.

From Equation, the magnification of the relay imaging group is −0.417 at the short focal length and −0.833 at the long focal length. A two-step zoom design with a three-component full-motion structure is used, and the zoom relay imaging group requires compensation for the residual aberration of the front concentric imaging group to improve the imaging quality of the system. The final design configuration of the zoom relay imaging group is illustrated in Figure 13. When the magnification of the relay imaging group is −0.417, the entire system is still an image-space telecentric structure.

**Figure 14:**
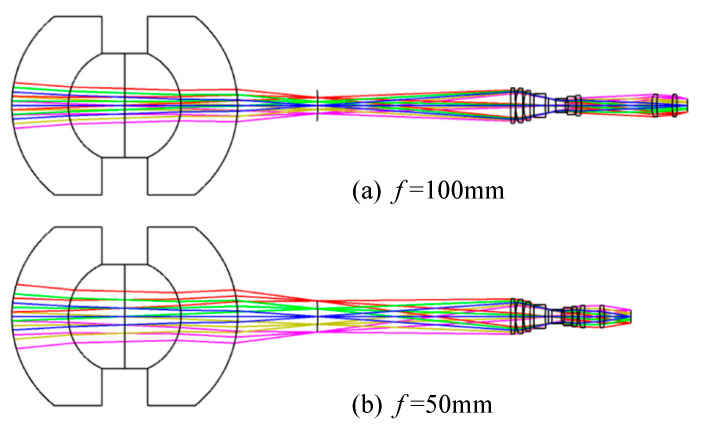
Configurations of the zoom concentric multi-scale optical system. (**a**) focal length *f* = 100 mm, (**b**) focal length *f* = 50 mm.

By combining the two components of the front concentric imaging group and the zoom relay imaging group, the final concentric multi-scale zoom optical system is obtained (as shown in Figure 14) which shows the concentric multi-scale optical system with a focal length of 100 mm and 50 mm.

#### 3.2.2. Overall Design of the Concentric Multi-Scale Zoom Optical System and Evaluation

**Figure 15:**
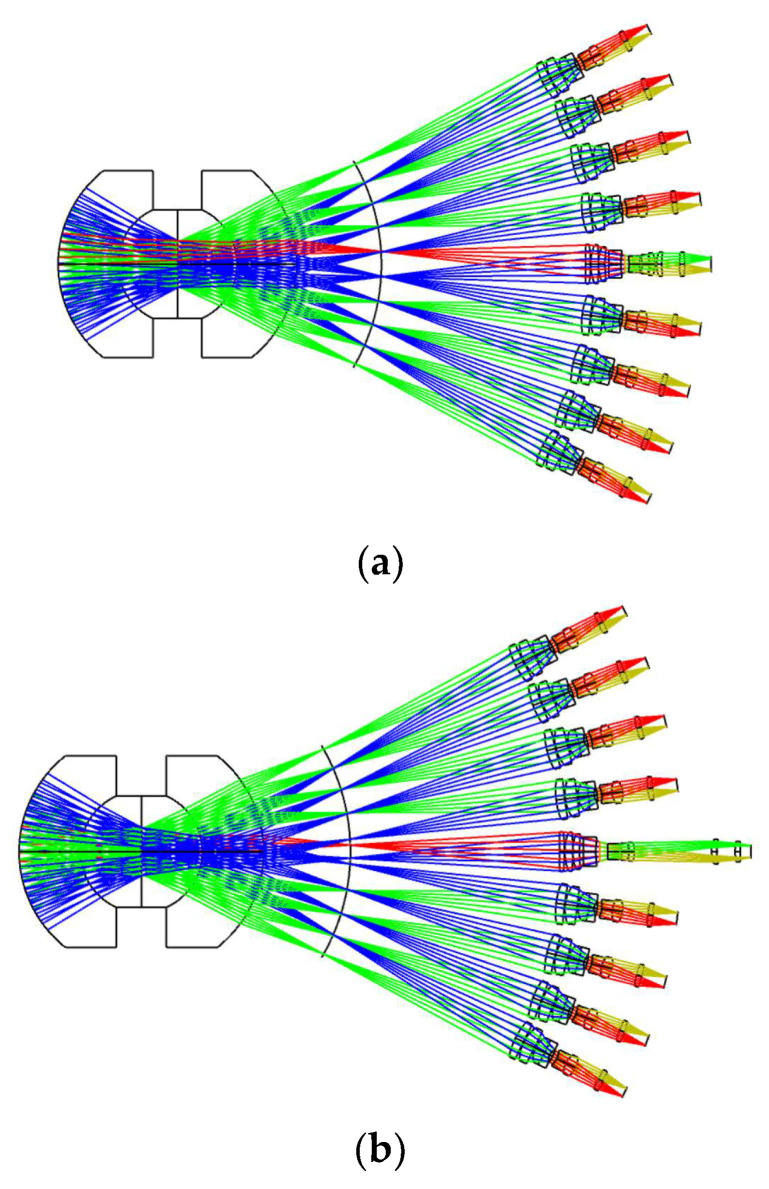
Configurations of the zoom concentric multi-scale optical system. (**a**) Central channel *f* = 50 mm, (**b**) Central channel *f* = 100 mm.

Based on the designs of three components: concentric imaging group, the image-space telecentric relay imaging group, and the zoom relay imaging group, the final entire concentric multi-scale zoom optical system (illustrated in Figure 15) is achieved. In Figure 15a, the focal length of each imaging channel is 50 mm, and the image-space telecentric structure is guaranteed; thus, the images can be stitched completely, and the images have the merit of high-precision stitching after focusing. In Figure 15b, the focal length of the center channel is 100 mm, the focal length of the remaining imaging channels is 50 mm, the images of the center channel are not engaged in stitching, and the images of the other optical channels can still be stitched normally. The center channel can perform high-resolution observation of the identified target of interest.

**Figure 16:**
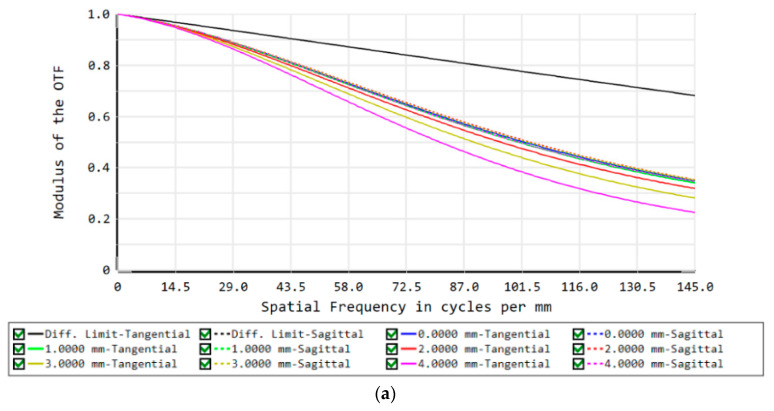

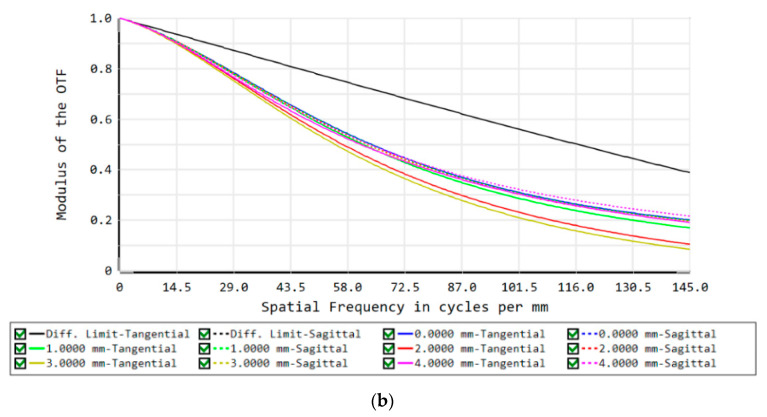
MTF curves of the concentric multi-scale zoom optical system. (**a**) Focal length *f* = 50 mm, (**b**) Focal length *f* = 100 mm.

**Figure 17:**
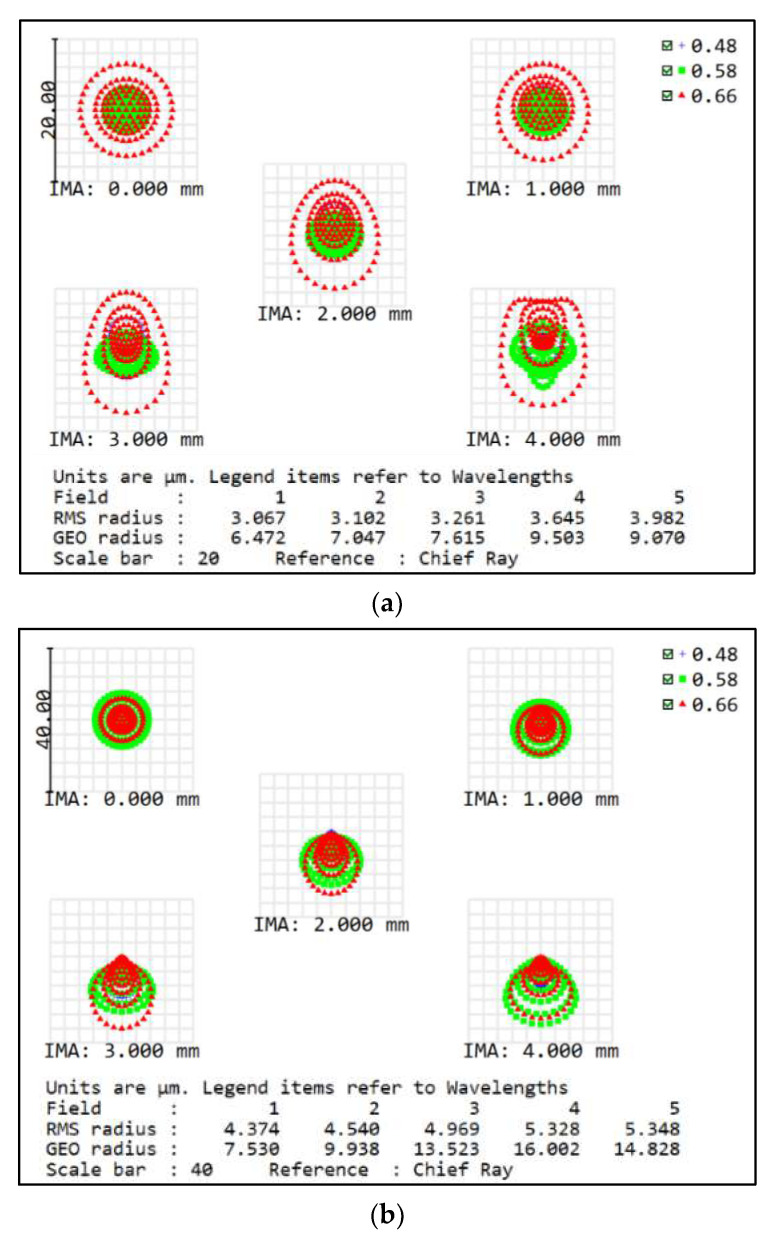
Spot diagrams of the concentric multi-scale zoom optical system. (**a**) Focal length *f* = 50 mm, (**b**) Focal length *f* = 100 mm.

The MTF curves of the concentric multi-scale zoom optical system (in Figure 16) show that the average MTF is greater than 0.3 (Nyquist frequency of 145 lp/mm) at a focal length of 50 mm, and the average MTF is greater than 0.15 (Nyquist frequency of 145 lp/mm) at a focal length of 100 mm. The spot diagrams of this system also are illustrated in Figure 17; the RMS radii of the spot diagram are small, showing good imaging quality irrespective of the focal length. The full FOV imaging quality has a good performance which indicates the availability of this system, and the final design demonstrates the feasibility and effectiveness of the joint design method proposed in this paper. This design will also be a valuable basis for the concentric multi-scale optical system design.

## 4. Conclusions

To address the accuracy and efficiency of the optical system for panoramic image stitching, this paper investigates the stability of the internal parameters of the concentric multi-scale optical system for high-resolution and point-to-point high-efficiency stitching imaging at different object distances. This paper proposes a design method for a concentric multi-scale zoom optical system based on wide object distance and high-precision imaging. The mechanism of image stitching misalignment is theoretically analyzed, and the design method of replacing the fixed-magnification relay imaging group with an image-space telecentric two-step zoom relay imaging group is proposed. Then, a telecentric multi-scale two-step zoom optical system is designed using the method proposed in this paper. When the focal length is 50 mm, the FOV of the concentric multi-scale optical system is 60° × 45°, and it has the advantage of high-precision imaging with wide object distance, which solves the misalignment problem of the stitched images after focusing. When the focal length of the center channel optical system is 100 mm, only the images made by the center channel optical system do not participate in image stitching, and the images of other channels still have the advantage of high-precision stitching. This paper proposes a concentric multi-scale zoom optical system based on wide object distance and high precision imaging, which has a broad application prospect in the field of a large FOV with high resolution.

However, there are two concerns with this paper’s concentric multi-scale zoom optical system based on wide-objective distance high-precision imaging as

(1) The number of lenses required for an image-space telecentric optical system will be higher compared to a non-image-space telecentric optical system.
(2) The imaging FOV of the concentric multi-scale optical system will vary during zooming, and the imaging FOV of adjacent cameras will no longer overlap, thus making image stitching impossible, so the 7 × 7 relay imaging group cannot all achieve zooming.

## Figures and Tables

**Table 1:** Design specifications of the concentric multi-scale optical system.

Parameters	Values
Focal length	50 mm
F-number	3.0
Working spectrum FOV Relay imaging camera	480–660 nm 60° × 45° 7 × 7
Nyquist MTF	145 lp/mm
Overlapping rate of FOV	5%

## Data Availability

Not applicable.

## References

[1] Golish D.R., Vera E.M., Kelly K.J., Gong Q., Jansen P.A., Hughes J.M., Kittle D.S., Brady D.J., Gehm M.E. (2012). Development of a scalable image formation pipeline for multiscale gigapixel photography. Opt. Express.

[2] Li J.H., Tan F.L., Zeng C.X., Ji Y.Q. (2021). Optical System Design of Wide-coverage and High-resolution Airborne Camera. Acta Opt. Sin..

[3] Brady D.J., Hagen N. (2009). Multiscale lens design. Opt. Express.

[4] Marks D.L., Son H.S., Kim J., Brady D.J. (2012). Engineering a gigapixel monocentric multiscale camera. Opt. Eng..

[5] Brady D.J., Gehm M.E., Stack R.A., Marks D.L., Kittle D.S., Golish D.R., Vera E.M., Feller S.D. (2012). Multiscale gigapixel photography. Nature.

[6] Johnson A., Mclaughlin P., Shaw J.M., Kim J., Hui S.S., Marks D.L., Brady D.J., Stack R.A. (2013). Optomechanical design of multiscale gigapixel digital camera. Appl. Opt..

[7] Liu F., Liu J.W., Shao X.P. (2020). Design of high integration and miniaturization concentric multiscale optical system. Opt. Precis. Eng..

[8] Yang W., Liu J.W., Han P.L., Shao X.P., Zhao X.M. (2019). Design of an infrared zoom imaging system based on concentric spherical lens with wide FOV and high resolution. J. Infrared Millim. Waves.

[9] Pang W., Brady D.J. (2017). Galilean monocentric multiscale optical systems. Opt. Express.

[10] Marks D.L., Tremblay E.J., Ford J.E., Brady D.J. (2011). Microcamera aperture scale in monocentric gigapixel cameras. Appl. Opt..

[11] Youn S.H., Son H.S., Marks D.L. (2014). Optical performance test and validation of microcameras in multiscale, gigapixel imagers. Opt. Express.

[12] Marks D.L., Llull P.R., Phillips Z., Anderson J.G., Feller S.D., Vera E.M., Son H.S., Youn S.H., Kim J., Gehm M.E. (2014). Characterization of the AWARE 10 two-gigapixel wide-field-of-view visible imager. Appl. Opt..

[13] Llull P., Bange L., Phillips Z., Davis K., Brandy D.J. (2015). Characterization of the AWARE 40 wide-field-of-view visible imager. Optica.

[14] Wu X.X. (2018). Design and Development of Wide FOV High Resolution Optical System Based on Multisacle Imaging Principle. Doctor’s Thesis.

[15] Chen H.Y., Miao F., Chen Y.J., Xiong Y.J., Chen T. (2021). A Hyperspectral Image Classification Method Using Multifeature Vectors and Optimized KELM. IEEE J. Sel. Top. Appl. Earth Obs. Remote Sens..

[16] Chen W., Liu Y., Wang Y.W., Sun J., Ji T., Zhao Q.L. (2021). Fast image stitching algorithm based on improved FAST-SURF. J. Appl. Opt..

[17] Liu T.T., Zhang J.L. (2018). Improved image stitching algorithm based on ORB features by UAV remote sensing. Comput. Eng. Appl..

[18] Yao R., Guo C., Deng W., Zhao H.M. (2021). A novel mathematical morphology spectrum entropy based on scale-adaptive techniques. ISA Trans..

[19] Li J.Y., Feng W.X., Liu F., Wei Y.Z., Shao X.P. (2021). Design of Airborne Multi-Scale Wide-Field-of-View and High-Resolution Imaging System. Acta Opt. Sin..

[20] Shen Y. (2018). Research on Super Large Field of View Optical Imaging Technology Based on Concentric Lens. Doctor’s Thesis.

[21] Lu W., Chen S., Xiong Y., Liu J. (2020). A single ball lens-based hybrid biomimetic fish eye/compound eye imaging system. Opt. Commun..

[22] Zhang K., Zhong X., Wang W., Meng Y., Ma C. (2019). High-efficiency calibration of star sensor based on two-dimensional Dammann grating. Opt. Eng..

[23] Zhang K. (2021). Research on the Space Target Measuring Optical System with High Precision and Large Field of View. Doctor’s Thesis.

